# (2.2.2-Cryptand)potassium tetra­carbonyl­cobaltate(−I)

**DOI:** 10.1107/S1600536814006758

**Published:** 2014-04-16

**Authors:** William W. Brennessel, John E. Ellis

**Affiliations:** a Department of Chemistry, 207 Pleasant Street SE, University of Minnesota, Minneapolis, MN 55455, USA

## Abstract

The title salt, [K(C_18_H_36_N_2_O_6_)][Co(CO)_4_], is an example of a classical carbonyl­metalate. The asymmetric unit contains one cation and one tetrahedral anion, both in general positions. Based on comparison of the four carbonyl C—O bond lengths and C—Co—C angles, the anion is unperturbed by the cation, which is normal for an alkali metal fully encased by a cryptand cage.

## Related literature   

For a survey of metal carbonyl anions, see: Ellis (2003 ▶). For the synthesis of the precursor bis­(anthracene)cobaltate, see: Brennessel *et al.* (2002 ▶). For an in-depth discussion of the perturbations of the title anion by cations in various solvents, as measured by IR spectroscopy, see: Edgell & Lyford (1971 ▶). For a description of the Cambridge Structural Database, see: Allen (2002 ▶). 
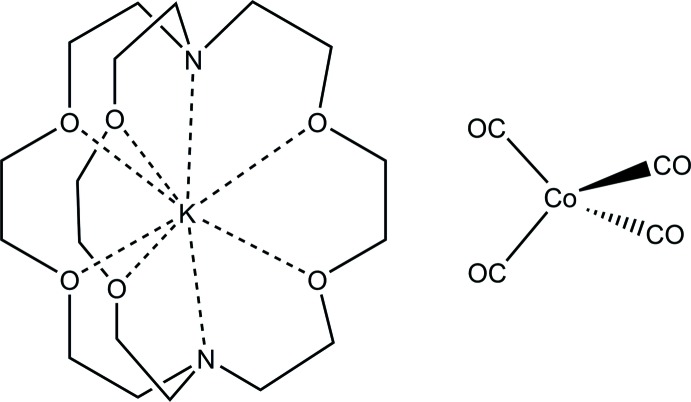


## Experimental   

### 

#### Crystal data  

[K(C_18_H_36_N_2_O_6_)][Co(CO)_4_]
*M* *_r_* = 586.56
Monoclinic, 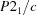

*a* = 9.3611 (18) Å
*b* = 12.022 (2) Å
*c* = 25.358 (5) Å
β = 91.536 (4)°
*V* = 2852.8 (9) Å^3^
*Z* = 4
Mo *K*α radiation
μ = 0.80 mm^−1^
*T* = 173 K
0.30 × 0.19 × 0.14 mm

#### Data collection  

Bruker SMART CCD platform diffractometer
Absorption correction: multi-scan (*SADABS*; Sheldrick, 2012 ▶) *T*_min_ = 0.521, *T*_max_ = 0.745
14351 measured reflections
5046 independent reflections
3551 reflections with *I* > 2σ(*I*)
*R*_int_ = 0.058

#### Refinement  

*R*[*F* ^2^ > 2σ(*F* ^2^)] = 0.040
*wR*(*F* ^2^) = 0.093
*S* = 0.92
5046 reflections
325 parameters
H-atom parameters constrained
Δρ_max_ = 0.39 e Å^−3^
Δρ_min_ = −0.23 e Å^−3^

### 

Data collection: *SMART* (Bruker, 2003 ▶); cell refinement: *SAINT* (Bruker, 2003 ▶); data reduction: *SAINT*; program(s) used to solve structure: *SIR97* (Altomare *et al.*, 1999 ▶); program(s) used to refine structure: *SHELXL2014* (Sheldrick, 2008 ▶); molecular graphics: *SHELXTL* (Sheldrick, 2008 ▶); software used to prepare material for publication: *SHELXTL*.

## Supplementary Material

Crystal structure: contains datablock(s) I, global. DOI: 10.1107/S1600536814006758/nk2222sup1.cif

Structure factors: contains datablock(s) I. DOI: 10.1107/S1600536814006758/nk2222Isup2.hkl

CCDC reference: 993915

Additional supporting information:  crystallographic information; 3D view; checkCIF report

## Figures and Tables

**Table 1:** Selected bond lengths (Å)

Co1—C4	1.762 (3)
Co1—C3	1.763 (4)
Co1—C1	1.767 (3)
Co1—C2	1.770 (4)

**Fig. 1.**
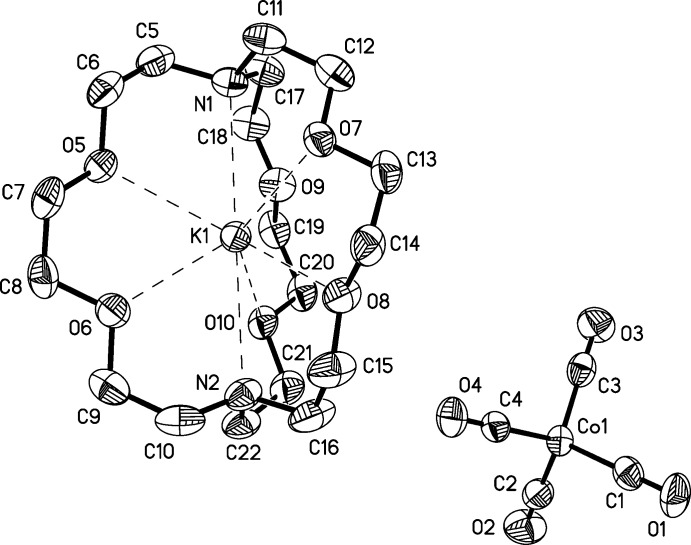
The structure of the title salt, showing the atom numbering. Displacement ellipsoids are drawn at the 50% probability level.

## supplementary crystallographic information

### 1. Comment

The title salt (Fig. 1) contains the classical metal carbonyl anion (Ellis, 2003) tetracarbonylcobaltate, which has now appeared in the Cambridge Structural Database (CSD, Version 5.35, update No. 2, Febuary 2014, Allen, 2002) 86 times, 70 for which it serves simply as an anion of modest bulk, as in the title salt. In eleven instances it is part of an alkali metal or thallium network, and in the remaining five occurrences it is coordinated through an oxygen atom to a transition metal or lanthanide.

Because the cobaltate is an unperturbed anion in this salt, the IR stretch is very strong and without features, as expected for a tetrahedron. In contrast, the very strong IR stretch of K[Co(CO)_4_] in instances in which there can be monodentate or tridentate K···O interactions has a shoulder that is due to symmetry reduction from *T_d_* to *C_3v_* (Edgell & Lyford, 1971).

### 2. Experimental

Argon was removed under vacuum from a deep pinkish-red solution of [K([2.2.2]cryptand)][Co(η^4^-C_14_H_10_)_2_]·0.5THF (Brennessel *et al.*, 2002) stirring in tetrahydrofuran (THF) at room temperature. Carbon monoxide (1 atm) was added, and the solution became immediately colorless. After a few minutes, the carbon monoxide and most of the solvent were removed under vacuum. Argon was reintroduced and diethyl ether was added to extract the anthracene and to precipitate the product. After filtering, washing (diethyl ether), and drying, the colorless salt was obtained in quantitative yield.

IR (νCO, THF, cm^-1^): 1892 *vs*; IR (νCO, Nujol mull, cm^-1^): 1878 *vs* br; ^59^Co NMR (71.15 MHz, CD_3_CN, 293 K, external reference 0.1 *M* K_3_[Co(CN)_6_] in D_2_O at 0.0 p.p.m., δ, p.p.m.): -3015.7 (*s*). Colorless blocks were grown from a pentane-layered THF solution at 273 K.

### 3. Refinement

All H atoms were placed geometrically and treated as riding atoms: C—H = 0.99 Å with *U*_iso(_H) = 1.2*U*_eq_(C).

### Crystal data

**Table:**

[K(C_18_H_36_N_2_O_6_)][Co(CO)_4_]	*F*(000) = 1232
*M**_r_* = 586.56	*D*_x_ = 1.366 Mg m^−^^3^
Monoclinic, *P*2_1_/*c*	Mo *K*α radiation, λ = 0.71073 Å
*a* = 9.3611 (18) Å	Cell parameters from 3996 reflections
*b* = 12.022 (2) Å	θ = 2.3–24.6°
*c* = 25.358 (5) Å	µ = 0.80 mm^−^^1^
β = 91.536 (4)°	*T* = 173 K
*V* = 2852.8 (9) Å^3^	Block, colorless
*Z* = 4	0.30 × 0.19 × 0.14 mm

### Data collection

**Table:**

Bruker SMART CCD platform diffractometer	3551 reflections with *I* > 2σ(*I*)
Radiation source: normal-focus sealed tube	*R*_int_ = 0.058
ω scans	θ_max_ = 25.1°, θ_min_ = 1.6°
Absorption correction: multi-scan (*SADABS*; Sheldrick, 2012)	*h* = −11→11
*T*_min_ = 0.521, *T*_max_ = 0.745	*k* = −11→14
14351 measured reflections	*l* = −29→30
5046 independent reflections	

### Refinement

**Table:**

Refinement on *F*^2^	Primary atom site location: structure-invariant direct methods
Least-squares matrix: full	Secondary atom site location: difference Fourier map
*R*[*F*^2^ > 2σ(*F*^2^)] = 0.040	Hydrogen site location: inferred from neighbouring sites
*wR*(*F*^2^) = 0.093	H-atom parameters constrained
*S* = 0.92	*w* = 1/[σ^2^(*F*_o_^2^) + (0.0428*P*)^2^] where *P* = (*F*_o_^2^ + 2*F*_c_^2^)/3
5046 reflections	(Δ/σ)_max_ < 0.001
325 parameters	Δρ_max_ = 0.39 e Å^−^^3^
0 restraints	Δρ_min_ = −0.23 e Å^−^^3^

### Special details

**Table:**

Geometry. All e.s.d.'s (except the e.s.d. in the dihedral angle between two l.s. planes) are estimated using the full covariance matrix. The cell e.s.d.'s are taken into account individually in the estimation of e.s.d.'s in distances, angles and torsion angles; correlations between e.s.d.'s in cell parameters are only used when they are defined by crystal symmetry. An approximate (isotropic) treatment of cell e.s.d.'s is used for estimating e.s.d.'s involving l.s. planes.

### Fractional atomic coordinates and isotropic or equivalent isotropic displacement parameters (Å^2^)

**Table:**

	*x*	*y*	*z*	*U*_iso_*/*U*_eq_	
Co1	0.03175 (4)	0.80932 (3)	0.38562 (2)	0.03777 (12)	
C1	0.1014 (3)	0.8470 (2)	0.44858 (12)	0.0452 (7)	
O1	0.1456 (3)	0.8706 (2)	0.48980 (8)	0.0653 (6)	
C2	−0.1456 (4)	0.8597 (3)	0.37998 (11)	0.0489 (8)	
O2	−0.2606 (3)	0.8925 (2)	0.37675 (10)	0.0736 (7)	
C3	0.0336 (3)	0.6631 (3)	0.38061 (11)	0.0462 (7)	
O3	0.0358 (2)	0.56731 (19)	0.37816 (9)	0.0651 (6)	
C4	0.1381 (3)	0.8663 (2)	0.33608 (11)	0.0436 (7)	
O4	0.2078 (3)	0.90302 (17)	0.30383 (8)	0.0627 (6)	
K1	0.37979 (6)	0.39030 (5)	0.37685 (2)	0.03466 (15)	
N1	0.2667 (2)	0.17944 (18)	0.42354 (9)	0.0408 (6)	
C5	0.2462 (3)	0.1981 (2)	0.48042 (11)	0.0488 (8)	
H5A	0.3399	0.1921	0.4992	0.059*	
H5B	0.1837	0.1387	0.4940	0.059*	
C6	0.1812 (3)	0.3093 (3)	0.49271 (11)	0.0520 (8)	
H6A	0.0928	0.3201	0.4710	0.062*	
H6B	0.1558	0.3120	0.5303	0.062*	
O5	0.28056 (19)	0.39546 (15)	0.48182 (7)	0.0399 (5)	
C7	0.2248 (3)	0.5016 (2)	0.49582 (11)	0.0482 (8)	
H7A	0.2008	0.5019	0.5336	0.058*	
H7B	0.1364	0.5170	0.4748	0.058*	
C8	0.3322 (3)	0.5887 (2)	0.48574 (11)	0.0504 (8)	
H8A	0.2952	0.6621	0.4965	0.060*	
H8B	0.4208	0.5733	0.5067	0.060*	
O6	0.3626 (2)	0.58999 (15)	0.43112 (7)	0.0438 (5)	
C9	0.4473 (3)	0.6823 (2)	0.41678 (12)	0.0512 (8)	
H9A	0.5469	0.6716	0.4299	0.061*	
H9B	0.4096	0.7511	0.4328	0.061*	
C10	0.4439 (3)	0.6927 (2)	0.35785 (12)	0.0535 (8)	
H10A	0.3437	0.7042	0.3456	0.064*	
H10B	0.4988	0.7597	0.3481	0.064*	
C11	0.1280 (3)	0.1528 (2)	0.39757 (12)	0.0481 (8)	
H11A	0.0532	0.2005	0.4126	0.058*	
H11B	0.1034	0.0745	0.4052	0.058*	
C12	0.1278 (3)	0.1691 (2)	0.33879 (12)	0.0492 (8)	
H12A	0.2091	0.1285	0.3237	0.059*	
H12B	0.0383	0.1392	0.3227	0.059*	
O7	0.13939 (19)	0.28435 (15)	0.32733 (7)	0.0396 (5)	
C13	0.1274 (3)	0.3049 (3)	0.27216 (10)	0.0480 (8)	
H13A	0.0340	0.2776	0.2582	0.058*	
H13B	0.2040	0.2650	0.2537	0.058*	
C14	0.1397 (3)	0.4268 (3)	0.26271 (11)	0.0461 (7)	
H14A	0.1179	0.4437	0.2251	0.055*	
H14B	0.0707	0.4674	0.2845	0.055*	
O8	0.28281 (19)	0.46132 (16)	0.27640 (7)	0.0458 (5)	
C15	0.3056 (3)	0.5738 (3)	0.26203 (12)	0.0610 (9)	
H15A	0.2431	0.6231	0.2825	0.073*	
H15B	0.2822	0.5843	0.2241	0.073*	
C16	0.4602 (3)	0.6026 (3)	0.27332 (12)	0.0619 (9)	
H16A	0.5212	0.5508	0.2535	0.074*	
H16B	0.4786	0.6787	0.2603	0.074*	
C17	0.3671 (3)	0.0864 (2)	0.41663 (13)	0.0509 (8)	
H17A	0.3546	0.0569	0.3804	0.061*	
H17B	0.3429	0.0261	0.4414	0.061*	
C18	0.5219 (3)	0.1185 (3)	0.42583 (12)	0.0507 (8)	
H18A	0.5350	0.1519	0.4613	0.061*	
H18B	0.5831	0.0515	0.4241	0.061*	
O9	0.56202 (19)	0.19592 (16)	0.38686 (7)	0.0466 (5)	
C19	0.7118 (3)	0.2156 (2)	0.38849 (11)	0.0473 (8)	
H19A	0.7635	0.1451	0.3823	0.057*	
H19B	0.7417	0.2442	0.4237	0.057*	
C20	0.7477 (3)	0.2982 (2)	0.34723 (11)	0.0471 (8)	
H20A	0.8527	0.3074	0.3461	0.056*	
H20B	0.7129	0.2716	0.3123	0.056*	
O10	0.68266 (18)	0.40194 (15)	0.35896 (7)	0.0404 (5)	
C21	0.7229 (3)	0.4842 (2)	0.32179 (11)	0.0504 (8)	
H21A	0.6887	0.4625	0.2860	0.060*	
H21B	0.8284	0.4905	0.3216	0.060*	
C22	0.6590 (3)	0.5936 (2)	0.33652 (12)	0.0472 (7)	
H22A	0.6855	0.6102	0.3738	0.057*	
H22B	0.7005	0.6526	0.3144	0.057*	
N2	0.5018 (2)	0.5968 (2)	0.33005 (9)	0.0432 (6)	

### Atomic displacement parameters (Å^2^)

**Table:**

	*U*^11^	*U*^22^	*U*^33^	*U*^12^	*U*^13^	*U*^23^
Co1	0.0357 (2)	0.0434 (2)	0.0341 (2)	−0.00146 (18)	0.00036 (16)	0.00340 (17)
C1	0.0456 (19)	0.0426 (17)	0.0476 (18)	−0.0042 (14)	0.0054 (15)	0.0106 (14)
O1	0.0779 (17)	0.0789 (17)	0.0382 (12)	−0.0091 (13)	−0.0153 (11)	0.0002 (11)
C2	0.053 (2)	0.0490 (18)	0.0451 (17)	−0.0006 (16)	0.0038 (16)	−0.0033 (14)
O2	0.0462 (15)	0.0820 (19)	0.0924 (18)	0.0188 (13)	−0.0037 (13)	−0.0085 (14)
C3	0.0390 (18)	0.061 (2)	0.0384 (16)	−0.0029 (15)	−0.0038 (13)	−0.0001 (15)
O3	0.0783 (17)	0.0451 (14)	0.0711 (15)	−0.0066 (12)	−0.0143 (13)	−0.0077 (12)
C4	0.0494 (19)	0.0424 (17)	0.0386 (16)	0.0051 (14)	−0.0055 (14)	0.0010 (14)
O4	0.0755 (16)	0.0628 (15)	0.0510 (13)	−0.0019 (12)	0.0223 (12)	0.0182 (11)
K1	0.0300 (3)	0.0400 (3)	0.0340 (3)	−0.0031 (3)	0.0000 (2)	−0.0008 (3)
N1	0.0344 (13)	0.0383 (13)	0.0495 (14)	−0.0043 (11)	0.0000 (11)	0.0027 (11)
C5	0.0470 (19)	0.054 (2)	0.0457 (16)	−0.0079 (15)	0.0046 (14)	0.0111 (15)
C6	0.049 (2)	0.064 (2)	0.0440 (17)	−0.0022 (17)	0.0151 (15)	0.0043 (16)
O5	0.0345 (11)	0.0454 (12)	0.0400 (10)	0.0021 (9)	0.0076 (8)	0.0007 (9)
C7	0.0464 (19)	0.059 (2)	0.0395 (16)	0.0137 (16)	0.0123 (14)	−0.0012 (15)
C8	0.061 (2)	0.0461 (18)	0.0443 (17)	0.0148 (16)	0.0032 (15)	−0.0119 (14)
O6	0.0429 (12)	0.0415 (11)	0.0472 (11)	−0.0025 (9)	0.0035 (9)	−0.0040 (9)
C9	0.0429 (18)	0.0385 (17)	0.072 (2)	−0.0015 (14)	0.0036 (16)	−0.0074 (16)
C10	0.0372 (18)	0.0449 (18)	0.078 (2)	−0.0054 (15)	0.0016 (16)	0.0192 (17)
C11	0.0353 (18)	0.0441 (17)	0.065 (2)	−0.0064 (14)	−0.0034 (15)	0.0063 (15)
C12	0.0429 (19)	0.0422 (19)	0.0618 (19)	−0.0042 (14)	−0.0080 (15)	−0.0065 (15)
O7	0.0400 (11)	0.0412 (11)	0.0373 (10)	−0.0036 (9)	−0.0043 (8)	−0.0040 (8)
C13	0.0459 (19)	0.061 (2)	0.0364 (15)	−0.0009 (15)	−0.0063 (13)	−0.0111 (15)
C14	0.0403 (18)	0.063 (2)	0.0344 (15)	−0.0039 (15)	−0.0097 (13)	−0.0002 (14)
O8	0.0368 (12)	0.0596 (13)	0.0406 (11)	−0.0091 (9)	−0.0085 (9)	0.0089 (10)
C15	0.053 (2)	0.074 (2)	0.0551 (19)	−0.0184 (18)	−0.0204 (16)	0.0281 (17)
C16	0.053 (2)	0.079 (2)	0.0531 (19)	−0.0237 (18)	−0.0061 (16)	0.0281 (18)
C17	0.049 (2)	0.0367 (17)	0.067 (2)	−0.0009 (14)	−0.0008 (16)	0.0014 (15)
C18	0.0392 (18)	0.0495 (19)	0.0630 (19)	0.0065 (15)	−0.0036 (15)	0.0050 (16)
O9	0.0327 (12)	0.0509 (12)	0.0559 (12)	0.0042 (9)	−0.0032 (9)	0.0025 (10)
C19	0.0298 (16)	0.057 (2)	0.0546 (18)	0.0092 (14)	−0.0013 (14)	−0.0140 (15)
C20	0.0313 (16)	0.061 (2)	0.0489 (17)	0.0050 (14)	0.0073 (13)	−0.0147 (15)
O10	0.0291 (10)	0.0511 (12)	0.0413 (10)	0.0019 (9)	0.0092 (8)	−0.0063 (9)
C21	0.0365 (17)	0.065 (2)	0.0501 (18)	−0.0096 (15)	0.0127 (14)	−0.0038 (16)
C22	0.0329 (17)	0.057 (2)	0.0522 (17)	−0.0137 (14)	0.0038 (14)	0.0079 (15)
N2	0.0323 (13)	0.0560 (16)	0.0413 (13)	−0.0086 (12)	−0.0004 (11)	0.0091 (12)

### Geometric parameters (Å, º)

**Table:**

Co1—C4	1.762 (3)	C11—H11A	0.9900
Co1—C3	1.763 (4)	C11—H11B	0.9900
Co1—C1	1.767 (3)	C12—O7	1.420 (3)
Co1—C2	1.770 (4)	C12—H12A	0.9900
C1—O1	1.149 (3)	C12—H12B	0.9900
C2—O2	1.147 (3)	O7—C13	1.422 (3)
C3—O3	1.153 (4)	C13—C14	1.490 (4)
C4—O4	1.148 (3)	C13—H13A	0.9900
K1—O6	2.7741 (19)	C13—H13B	0.9900
K1—O8	2.8137 (18)	C14—O8	1.436 (3)
K1—O5	2.8433 (18)	C14—H14A	0.9900
K1—O7	2.8481 (19)	C14—H14B	0.9900
K1—O10	2.8866 (19)	O8—C15	1.418 (3)
K1—O9	2.900 (2)	C15—C16	1.508 (4)
K1—N2	2.991 (2)	C15—H15A	0.9900
K1—N1	3.003 (2)	C15—H15B	0.9900
N1—C17	1.474 (4)	C16—N2	1.482 (4)
N1—C11	1.475 (3)	C16—H16A	0.9900
N1—C5	1.477 (3)	C16—H16B	0.9900
C5—C6	1.505 (4)	C17—C18	1.512 (4)
C5—H5A	0.9900	C17—H17A	0.9900
C5—H5B	0.9900	C17—H17B	0.9900
C6—O5	1.424 (3)	C18—O9	1.416 (3)
C6—H6A	0.9900	C18—H18A	0.9900
C6—H6B	0.9900	C18—H18B	0.9900
O5—C7	1.427 (3)	O9—C19	1.421 (3)
C7—C8	1.478 (4)	C19—C20	1.488 (4)
C7—H7A	0.9900	C19—H19A	0.9900
C7—H7B	0.9900	C19—H19B	0.9900
C8—O6	1.421 (3)	C20—O10	1.423 (3)
C8—H8A	0.9900	C20—H20A	0.9900
C8—H8B	0.9900	C20—H20B	0.9900
O6—C9	1.417 (3)	O10—C21	1.424 (3)
C9—C10	1.499 (4)	C21—C22	1.497 (4)
C9—H9A	0.9900	C21—H21A	0.9900
C9—H9B	0.9900	C21—H21B	0.9900
C10—N2	1.464 (4)	C22—N2	1.477 (3)
C10—H10A	0.9900	C22—H22A	0.9900
C10—H10B	0.9900	C22—H22B	0.9900
C11—C12	1.503 (4)		
			
C4—Co1—C3	109.24 (13)	C12—C11—H11A	109.0
C4—Co1—C1	110.01 (13)	N1—C11—H11B	109.0
C3—Co1—C1	108.48 (13)	C12—C11—H11B	109.0
C4—Co1—C2	110.86 (13)	H11A—C11—H11B	107.8
C3—Co1—C2	110.27 (14)	O7—C12—C11	109.4 (2)
C1—Co1—C2	107.94 (13)	O7—C12—H12A	109.8
O1—C1—Co1	179.1 (3)	C11—C12—H12A	109.8
O2—C2—Co1	179.4 (3)	O7—C12—H12B	109.8
O3—C3—Co1	178.9 (3)	C11—C12—H12B	109.8
O4—C4—Co1	179.7 (3)	H12A—C12—H12B	108.2
O6—K1—O8	99.49 (6)	C12—O7—C13	111.5 (2)
O6—K1—O5	59.46 (5)	C12—O7—K1	114.18 (15)
O8—K1—O5	137.33 (6)	C13—O7—K1	113.37 (15)
O6—K1—O7	123.37 (6)	O7—C13—C14	109.0 (2)
O8—K1—O7	60.31 (5)	O7—C13—H13A	109.9
O5—K1—O7	98.53 (5)	C14—C13—H13A	109.9
O6—K1—O10	96.11 (5)	O7—C13—H13B	109.9
O8—K1—O10	97.90 (5)	C14—C13—H13B	109.9
O5—K1—O10	119.53 (5)	H13A—C13—H13B	108.3
O7—K1—O10	136.07 (5)	O8—C14—C13	108.8 (2)
O6—K1—O9	134.08 (6)	O8—C14—H14A	109.9
O8—K1—O9	119.90 (6)	C13—C14—H14A	109.9
O5—K1—O9	98.15 (5)	O8—C14—H14B	109.9
O7—K1—O9	97.71 (6)	C13—C14—H14B	109.9
O10—K1—O9	58.33 (5)	H14A—C14—H14B	108.3
O6—K1—N2	60.43 (6)	C15—O8—C14	111.1 (2)
O8—K1—N2	60.42 (6)	C15—O8—K1	118.35 (16)
O5—K1—N2	119.41 (6)	C14—O8—K1	114.04 (14)
O7—K1—N2	120.12 (6)	O8—C15—C16	108.7 (3)
O10—K1—N2	60.68 (6)	O8—C15—H15A	110.0
O9—K1—N2	118.26 (6)	C16—C15—H15A	110.0
O6—K1—N1	120.61 (6)	O8—C15—H15B	110.0
O8—K1—N1	120.30 (6)	C16—C15—H15B	110.0
O5—K1—N1	61.43 (6)	H15A—C15—H15B	108.3
O7—K1—N1	60.89 (6)	N2—C16—C15	113.5 (2)
O10—K1—N1	117.46 (6)	N2—C16—H16A	108.9
O9—K1—N1	59.87 (6)	C15—C16—H16A	108.9
N2—K1—N1	178.13 (7)	N2—C16—H16B	108.9
C17—N1—C11	109.8 (2)	C15—C16—H16B	108.9
C17—N1—C5	109.3 (2)	H16A—C16—H16B	107.7
C11—N1—C5	109.4 (2)	N1—C17—C18	113.5 (2)
C17—N1—K1	111.16 (16)	N1—C17—H17A	108.9
C11—N1—K1	108.83 (16)	C18—C17—H17A	108.9
C5—N1—K1	108.26 (16)	N1—C17—H17B	108.9
N1—C5—C6	113.6 (2)	C18—C17—H17B	108.9
N1—C5—H5A	108.8	H17A—C17—H17B	107.7
C6—C5—H5A	108.8	O9—C18—C17	109.3 (2)
N1—C5—H5B	108.8	O9—C18—H18A	109.8
C6—C5—H5B	108.8	C17—C18—H18A	109.8
H5A—C5—H5B	107.7	O9—C18—H18B	109.8
O5—C6—C5	109.7 (2)	C17—C18—H18B	109.8
O5—C6—H6A	109.7	H18A—C18—H18B	108.3
C5—C6—H6A	109.7	C18—O9—C19	111.7 (2)
O5—C6—H6B	109.7	C18—O9—K1	115.11 (15)
C5—C6—H6B	109.7	C19—O9—K1	116.48 (16)
H6A—C6—H6B	108.2	O9—C19—C20	109.4 (2)
C6—O5—C7	110.9 (2)	O9—C19—H19A	109.8
C6—O5—K1	113.40 (15)	C20—C19—H19A	109.8
C7—O5—K1	112.52 (14)	O9—C19—H19B	109.8
O5—C7—C8	109.6 (2)	C20—C19—H19B	109.8
O5—C7—H7A	109.8	H19A—C19—H19B	108.2
C8—C7—H7A	109.8	O10—C20—C19	109.4 (2)
O5—C7—H7B	109.8	O10—C20—H20A	109.8
C8—C7—H7B	109.8	C19—C20—H20A	109.8
H7A—C7—H7B	108.2	O10—C20—H20B	109.8
O6—C8—C7	109.3 (2)	C19—C20—H20B	109.8
O6—C8—H8A	109.8	H20A—C20—H20B	108.2
C7—C8—H8A	109.8	C20—O10—C21	110.4 (2)
O6—C8—H8B	109.8	C20—O10—K1	114.71 (15)
C7—C8—H8B	109.8	C21—O10—K1	114.61 (15)
H8A—C8—H8B	108.3	O10—C21—C22	109.3 (2)
C9—O6—C8	112.7 (2)	O10—C21—H21A	109.8
C9—O6—K1	120.67 (15)	C22—C21—H21A	109.8
C8—O6—K1	119.31 (16)	O10—C21—H21B	109.8
O6—C9—C10	108.9 (2)	C22—C21—H21B	109.8
O6—C9—H9A	109.9	H21A—C21—H21B	108.3
C10—C9—H9A	109.9	N2—C22—C21	113.5 (2)
O6—C9—H9B	109.9	N2—C22—H22A	108.9
C10—C9—H9B	109.9	C21—C22—H22A	108.9
H9A—C9—H9B	108.3	N2—C22—H22B	108.9
N2—C10—C9	114.6 (2)	C21—C22—H22B	108.9
N2—C10—H10A	108.6	H22A—C22—H22B	107.7
C9—C10—H10A	108.6	C10—N2—C22	110.3 (2)
N2—C10—H10B	108.6	C10—N2—C16	109.9 (2)
C9—C10—H10B	108.6	C22—N2—C16	110.1 (2)
H10A—C10—H10B	107.6	C10—N2—K1	108.21 (15)
N1—C11—C12	113.0 (2)	C22—N2—K1	108.94 (16)
N1—C11—H11A	109.0	C16—N2—K1	109.33 (17)
			
C17—N1—C5—C6	163.4 (2)	K1—O8—C15—C16	49.7 (3)
C11—N1—C5—C6	−76.4 (3)	O8—C15—C16—N2	−63.7 (4)
K1—N1—C5—C6	42.1 (3)	C11—N1—C17—C18	161.4 (2)
N1—C5—C6—O5	−68.5 (3)	C5—N1—C17—C18	−78.5 (3)
C5—C6—O5—C7	−176.8 (2)	K1—N1—C17—C18	41.0 (3)
C5—C6—O5—K1	55.4 (3)	N1—C17—C18—O9	−65.0 (3)
C6—O5—C7—C8	178.1 (2)	C17—C18—O9—C19	−170.7 (2)
K1—O5—C7—C8	−53.7 (3)	C17—C18—O9—K1	53.5 (3)
O5—C7—C8—O6	61.3 (3)	C18—O9—C19—C20	−179.4 (2)
C7—C8—O6—C9	170.6 (2)	K1—O9—C19—C20	−44.3 (3)
C7—C8—O6—K1	−38.9 (3)	O9—C19—C20—O10	64.3 (3)
C8—O6—C9—C10	−166.3 (2)	C19—C20—O10—C21	176.4 (2)
K1—O6—C9—C10	43.8 (3)	C19—C20—O10—K1	−52.4 (3)
O6—C9—C10—N2	−61.4 (3)	C20—O10—C21—C22	−177.2 (2)
C17—N1—C11—C12	−78.8 (3)	K1—O10—C21—C22	51.4 (3)
C5—N1—C11—C12	161.2 (2)	O10—C21—C22—N2	−68.2 (3)
K1—N1—C11—C12	43.0 (3)	C9—C10—N2—C22	−72.8 (3)
N1—C11—C12—O7	−68.3 (3)	C9—C10—N2—C16	165.6 (2)
C11—C12—O7—C13	−175.0 (2)	C9—C10—N2—K1	46.3 (3)
C11—C12—O7—K1	54.9 (3)	C21—C22—N2—C10	164.9 (2)
C12—O7—C13—C14	179.8 (2)	C21—C22—N2—C16	−73.7 (3)
K1—O7—C13—C14	−49.7 (3)	C21—C22—N2—K1	46.2 (3)
O7—C13—C14—O8	67.6 (3)	C15—C16—N2—C10	−74.6 (3)
C13—C14—O8—C15	172.7 (2)	C15—C16—N2—C22	163.7 (3)
C13—C14—O8—K1	−50.5 (2)	C15—C16—N2—K1	44.1 (3)
C14—O8—C15—C16	−175.5 (2)		
